# Gender Difference in the Associations among Heavy Metals with Red Blood Cell Hemogram

**DOI:** 10.3390/ijerph19010189

**Published:** 2021-12-24

**Authors:** Chao-Hsin Huang, Chih-Wen Wang, Huang-Chi Chen, Hung-Pin Tu, Szu-Chia Chen, Chih-Hsing Hung, Chao-Hung Kuo

**Affiliations:** 1 Department of Post Baccalaureate Medicine, Kaohsiung Medical University, Kaohsiung 807, Taiwan; dragonc09931227@gmail.com; 2 Department of Internal Medicine, Kaohsiung Municipal Siaogang Hospital, Kaohsiung Medical University, Kaohsiung 812, Taiwan; chinwin.wang@gmail.com (C.-W.W.); 890080@kmhk.org.tw (H.-C.C.); kjh88kmu@gmail.com (C.-H.K.); 3 Department of Internal Medicine, Division of Hepatobiliary, Kaohsiung Medical University Hospital, Kaohsiung Medical University, Kaohsiung 807, Taiwan; 4 Department of Internal Medicine, Division of Pulmonary and Critical Care Medicine, Kaohsiung Medical University Hospital, Kaohsiung Medical University, Kaohsiung 807, Taiwan; 5 Department of Public Health and Environmental Medicine, School of Medicine, College of Medicine, Kaohsiung Medical University, Kaohsiung 807, Taiwan; p915013@kmu.edu.tw; 6 Department of Internal Medicine, Division of Nephrology, Kaohsiung Medical University Hospital, Kaohsiung Medical University, Kaohsiung 807, Taiwan; 7 Faculty of Medicine, College of Medicine, Kaohsiung Medical University, Kaohsiung 807, Taiwan; 8 Research Center for Environmental Medicine, Kaohsiung Medical University, Kaohsiung 807, Taiwan; 9 Department of Pediatrics, Kaohsiung Medical University Hospital, Kaohsiung Medical University, Kaohsiung 807, Taiwan; 10 Department of Pediatrics, Kaohsiung Municipal Siaogang Hospital, Kaohsiung Medical University, Kaohsiung 812, Taiwan; 11 Department of Internal Medicine, Division of Gastroenterology, Kaohsiung Medical University Hospital, Kaohsiung Medical University, Kaohsiung 807, Taiwan

**Keywords:** heavy metal, hemoglobin, mean corpuscular volume, mean corpuscular hemoglobin concentration

## Abstract

This study aimed to investigate gender differences in the association between heavy metals and hemograms including hemoglobin (Hgb), mean corpuscular volume (MCV) and mean corpuscular hemoglobin concentration (MCHC). A health survey of 2447 participants was conducted in southern Taiwan between June 2016 and September 2018. Seven heavy metals were measured: blood lead (Pb), urine nickel (Ni), urine chromium (Cr), urine manganese, urine arsenic (As), urine copper and urine cadmium (Cd). The results show that in females, Pb and Ni were significantly negatively associated with Hgb. In addition, As and Cd were significantly positively, and Pb and Ni were significantly negatively, associated with MCV, in males and females, respectively. The interactions between gender and Ni and gender and Cd in MCV were statistically significant. Further, Pb, in males, and Pb, Ni and Cr, in females, were significantly negatively associated with MCHC. In conclusion, in females, associations of red blood cell (RBC) hemograms with heavy metals such as Pb and Ni were found. In males, heavy metals such as Pb, As and Cd were found to associate with RBC hemograms. Further research is warranted to discuss the mechanism behind these associations.

## 1. Introduction

The detrimental effects of industrial waste have been investigated, including changes in immune function and metabolism disturbances leading to comorbidities [1,2,3]. Animal studies pointed out the effects of heavy metals on hemogram parameters [4]. For instance, hemogram imbalance such as decreased levels of hemoglobin (Hgb) and hematocrit was observed by Vinodhini et al. using a fish model [4]. Thus, investigating heavy metal pollution is critical for early diagnosis and treatment to avoid consequent comorbidities. Hemogram parameters, on the other hand, provide information regarding human health. Several studies showed the importance of hemogram parameters as predictors or indicators of dementia [5], primary ovarian insufficiency [6] and type 2 diabetes mellitus [3]. However, few studies have investigated the association between heavy metal pollution and hemogram parameters.

In addition, gender differences in red blood cell (RBC) hemograms have been observed in several studies [7,8]. For example, males and females have been found to have a 12% difference in Hgb levels [8]. Some hemogram differences are constitutive, although some have been correlated with hormone effects on erythropoiesis [9,10]. Other factors such as bone marrow function also affect erythropoiesis, which may be correlated with heavy metal exposure [11]. Hemogram-related diseases such as iron deficiency anemia, anemia and thalassemia have been associated with a high cadmium (Cd) level [2,12]. Since few studies have investigated gender differences in this relationship, the aim of this study was to investigate gender differences in the effects of heavy metal exposure on RBC hemograms. Seven heavy metals were studied, including blood lead (Pb), urine nickel (Ni), urine chromium (Cr), urine manganese (Mn), urine arsenic (As), urine copper (Cu) and urine Cd, as well as RBC hemogram parameters including Hgb, mean corpuscular volume (MCV) and mean corpuscular hemoglobin concentration (MCHC).

## 2. Materials and Methods

### 2.1. Study Subjects

This study enrolled participants from the general population living in the south of Taiwan, all of whom provided informed consent before being enrolled into this study. The participants attended a health survey which was conducted from June 2016 to September 2018 and promoted through advertisements. All of the participants completed a physical examination conducted by an experienced physician and were asked about their medical histories, including diabetes and hypertension. During the examination, height, weight, systolic blood pressure [SBP] and diastolic blood pressure [DBP] were recorded. Study subjects were defined as having diabetes mellitus if the fasting blood glucose level was greater than 126 mg/dL or hypoglycemic agents were used to control blood glucose levels. Similarly, study patients were considered as having hypertension if the SBP was ≥140 mmHg or DBP ≥ 90 mmHg or anti-hypertensive drugs were prescribed. This study was approved by the Institutional Review Board of Kaohsiung Medical University Hospital (number: KMUHIRB-G(II)-20190011).

### 2.2. Laboratory, Medical and Demographic Data

In addition to the variables mentioned above, laboratory data (fasting glucose, triglycerides, total, high-density lipoprotein (HDL) and low-density lipoprotein (LDL) cholesterol, Hgb, MCV, MCHC, estimated glomerular filtration rate (eGFR) and uric acid) were also recorded at baseline. Laboratory data were measured from fasting blood samples using an autoanalyzer (Roche Diagnostics GmbH, D-68298 Mannheim COBAS Integra 400). Serum creatinine was measured by the compensated Jaffé (kinetic alkaline picrate) method in a Roche/Integra 400 Analyzer (Roche Diagnostics, Mannheim, Germany) using a calibrator traceable to isotope dilution mass spectrometry. The eGFR was calculated using the Chronic Kidney Disease Epidemiology Collaboration equation (CKD-EPI eGFR) [13]. Body mass index (BMI) was calculated as weight/height squared (kg/m^2^).

RBC hemograms were measured using the automatic blood analyzer XN-3000. The analytical measurement range of Hgb was 0.0–26.0 g/dL. The precisions of Hgb, MCV and MCHC were 1.0%, 1.0% and 2.0%, respectively. The accuracies of Hgb and MCV were within ±2.0% and ±3.0%, respectively.

### 2.3. Blood and Urine Heavy Metal Measurements

The following heavy metals were assessed in this study: urine Cd, Cu, As, Mn, Cr and Ni concentrations, and blood Pb concentration. All measurements were obtained by graphite furnace atomic absorption spectrometry (NexION 300, Perkin Elmer, Shelton, CT, USA) as previously reported by a clinical laboratory (Union Clinical Laboratory Taipei, Taiwan, CAP Number: 6979606) accredited by the College of American Pathologists. Daily internal and external quality control testing was performed to ensure the accuracy of the measurements.

The digestion methods used prior to the instrumental analysis were as follows:

1. Urine As, Cu: After shaking the clinical urine sample for 5 s, absorb the suspension, dilute 20X: 250 uL urine sample + 100 uL internal control + 4.65 mL 1.0% HNO_3_, shake and centrifuge at 3000× *g* rpm for 10 min.
2. Urine Mn, Ni, Cd: After shaking the clinical urine sample for 5 s, absorb the suspension and dilute it 20X: 250 uL urine sample + 100 uL internal control + 4.65 mL 0.5% HNO3 and centrifuge at 3000× *g* rpm for 10 min after shaking.
3. Urine Cr: After shaking the clinical urine sample for 5 s, absorb the suspension, dilute 20X: 250 uL urine sample + 100 uL internal control + 4.65 mL 0.5% (HNO_3_ + 0.5% TX-100), shake and centrifuge at 3000× *g* rpm for 10 min.

The LOQ of each heavy metal was as follows:

1. Blood Pb: 8.2 ug/dL;
2. Urine Cd: 0.5 ppb;
3. Urine Cu: 1.0 ppb;
4. Urine Ni: 1.0 ppb;
5. Urine As: 1.0 ppb;
6. Urine Cr: 0.2ppb;
7. Urine Mn: 0.5 ppb.

### 2.4. Statistical Analysis

Data are presented as percentages, means ± standard deviations or medians. Heavy metals and triglycerides are presented as 25th–75th percentiles. The chi-square test was used to compare categorical variables, and the independent t-test was used to compare continuous variables. All urine heavy metals in further analysis were adjusted for urine creatinine. Linear relationships between the dependent and independent variables and independence of observations were tested by using the Durbin–Watson statistic. Moreover, we used visual inspection of a plot of studentized residuals versus unstandardized predicted values to assess homoscedasticity and the residuals of the regression line. We checked the correlation coefficient by Pearson’s correlation and then used the variance inflation factor to assess multicollinearity. Univariate and multivariable linear regression analyses were used to identify associations between heavy metals and Hgb, MCV and MCHC in the male and female participants. Heavy metals and significant variables in the univariate analysis were further analyzed by multivariable analysis. Pearson’s correlation r between total cholesterol, HDL cholesterol and LDL cholesterol was greater than 0.7. Therefore, we excluded HDL-L and LDL-L in further multivariable analysis if total cholesterol existed. Interactions between gender and heavy metals in RBC hemograms were analyzed using a generalized linear model. Because the values of heavy metals presented an abnormal distribution, the natural logarithm was used for all heavy metal measurements. A *p*-value of < 0.05 was considered to indicate a statistically significant difference. All statistical analyses were performed using SPSS (version 19.0 for Windows, SPSS Inc., Chicago, IL, USA).

## 3. Results

The mean age of the 2447 participants (977 males and 1470 females) was 55.1 ± 13.2 years. A comparison of the clinical characteristics among the male and female participants is shown in Table 1. Compared to the male participants, the female participants had a lower prevalence rate of hypertension, lower BMI, lower DBP, lower fasting glucose, lower triglycerides, higher total cholesterol, higher HDL cholesterol, lower Hgb, lower MCV, lower MCHC and lower uric acid. Regarding heavy metals, the female participants had lower blood Pb, higher urine Ni, higher urine As, lower urine Cu and higher urine Cd. 

### 3.1. Associations between Heavy Metals and Hgb in the Male and Female Participants

Table 2 shows the associations between heavy metals and Hgb using univariable linear regression analysis by gender. Heavy metals and significant variables in the univariate analysis were further analyzed by multivariable analysis.

Table 3 shows the associations between heavy metals and Hgb using multivariable linear regression analysis by gender. In female participants, after adjusting for age, diabetes, systolic and diastolic blood pressures, log triglyceride, total cholesterol and uric acid (significant variables in Table 2, but excluding HDL cholesterol and LDL cholesterol due to multicollinearity), blood Pb and urine Ni were significantly negatively associated with Hgb.

However, no significant association was noted in the male participants.

The interactions between gender and As (*p* = 0.002) and gender and Cd in Hgb (*p* = 0.016) were statistically significant.

In our study, the Durbin–Watson statistic for our data was 1.964. The Durbin–Watson statistic can range from 0 to 4, which indicates that there is no correlation between residuals. 

There was homoscedasticity, as assessed by visual inspection of a plot of studentized residuals versus unstandardized predicted values and the residuals of the regression line, approximately normally distributed (mean ≅ 0, standard deviation ≅ 1).

### 3.2. Associations between Heavy Metals and MCV in the Male and Female Participants

Table 4 shows the associations between heavy metals and MCV using univariable linear regression analysis by gender. Heavy metals and significant variables in the univariate analysis were further analyzed by multivariable analysis.

Table 5 shows the associations between heavy metals and MCV using multivariable linear regression analysis by gender. After adjusting for age and eGFR (significant variables in Table 4), in male participants, urine As and urine Cd were significantly positively associated with MCV.

In addition, after adjusting for age, body mass index, SBP, total cholesterol and eGFR (significant variables in Table 4, but excluding HDL cholesterol and LDL cholesterol due to multicollinearity), in female participants, blood Pb and urine Ni were significantly negatively associated with MCV.

The interactions between gender and Ni (*p* = 0.013) and gender and Cd in MCV (*p* = 0.030) were statistically significant.

### 3.3. Associations between Heavy Metals and MCHC in the Male and Female Participants

Table 6 shows the associations between heavy metals and MCHC using univariable linear regression analysis by gender. Heavy metals and significant variables in the univariate analysis were further analyzed by multivariable analysis.

Table 7 shows the associations between heavy metals and MCHC using multivariable linear regression analysis by gender. After adjusting for age, systolic and diastolic blood pressures, log triglyceride, HDL cholesterol, eGFR and uric acid (significant variables in Table 6), in male participants, blood Pb was significantly negatively associated with MCHC. After adjusting for log triglyceride, total cholesterol and uric acid (significant variables in Table 6, but excluding LDL cholesterol due to multicollinearity), in female participants, blood Pb, urine Ni and urine Cr were significantly negatively associated with MCHC.

None of the interactions between gender and heavy metals in MCHC achieved significance.

### 3.4. Sensitivity Analyses

We further assessed the associations between heavy metals and RBC hemograms in the male (*n* = 646) and female (*n* = 1072) participants after excluding participants with diabetes or hypertension. In the female participants, blood Pb and urine Ni were significantly negatively associated with Hgb. Urine Cd was significantly positively associated with MCV in the male participants, and blood Pb and urine Ni were significantly negatively associated with MCV in the female participants. Urine Mn was significantly positively associated with MCHC, and blood Pb and urine Ni were significantly negatively associated with MCHC, in the female participants. 

## 4. Discussion

In this study, in females, Pb and Ni were negatively associated with Hgb, MCV and MCHC, and Cr was also negatively associated with MCHC. In males, As and Cd were positively associated with MCV, and Pb was negatively associated with MCHC. 

The first important finding is that female hemogram parameters were more susceptible to heavy metal poisoning such as Pb, Ni and Cr, but As affected males more than females. Previous studies reported gender dimorphism regarding heavy metal poisoning [14,15]. This sex dimorphism could relate to intrinsic sex-specific epigenetic mechanisms, hormone-regulated metabolism of heavy metals and iron-regulated metabolism [16,17]. These mechanisms can affect the absorption, retention and excretion of heavy metals, which consequently affects the level of heavy metals in different genders. For instance, females were more protected from As poisoning than males because arsenic methylation induced by estrogen decreased the level of toxicity in females [17]. Another hypothesis was that bone turnover increased as the estrogen level decreased, which released heavy metals stored in the bones. This resulted in the females’ susceptibility to heavy metal poisoning in certain physiological stages [18].

The second important finding of this study is that blood Pb was negatively associated with Hgb, MCV and MCHC in the female participants, and MCV in the males. The average Pb exposure in Taiwan is 160 μg/dL, higher than other countries such as Korea with blood Pb of 1.82 μg/dL [19]. The accumulation of Pb in the body has also been associated with the bone turnover rate, and high blood Pb has been associated with accelerated bone reabsorption in those with low estrogen levels [18]. One hypothesis is that low estrogen production after menopause is associated with bone demineralization, during which Pb is also released into the bloodstream [20]. The mechanism of Pb-induced anemia involves disruption of RBC membrane proteins, and an inverse relationship between the Pb level and RBC concentration has been found in previous studies [21,22]. In addition, Pb inhibits ferrochelatase, the terminal enzyme in the heme biosynthetic pathway, and causes microcytic anemia [23]. For example, Jacob et al. conducted a school survey of Pb exposure in 797 children in Germany and showed that MCV and MCH in girls decreased as the concentration of Pb in the blood increased [24]. In the present study, our results show that Pb was negatively correlated with MCV and MCHC in both the male and female participants, and negatively correlated with Hgb in the females. This result corresponds to a previous study, as microcytic anemia [23] was also observed in our study. In summary, we hypothesize that the Pb level increased as the estrogen level decreased, and the increased Pb level disrupted the RBC membrane and inhibited heme biosynthesis, which, in turn, led to microcytic anemia in females. 

The third important finding of this study is that urine Ni was negatively associated with Hgb, MCV and MCHC in the females. Furthermore, increased exposure to Ni was significantly related to a decreased MCV level in females. Ni toxicity has been shown to disrupt the reproductive axis and cause immune dysregulation and carcinogenicity, leading to cardiovascular disease, impaired lung function and dermatitis [25,26]. Ni has also been found to affect the activities and longevity of RBCs in healthy individuals due to the decreased thermostability of RBCs [27], oxidative stress and deoxyribonucleic acid (DNA) damage [25]. Disruption of iron hemostasis has also been associated with Ni toxicity [28]. The effects of Ni on erythropoiesis are still under investigation. Some studies have shown that Ni-induced hypoxemia leads to an increase in the number of RBCs, although other studies have shown opposite results [28,29,30]. In our study, Ni was negatively correlated with Hgb, MCV and MCHC in the female participants. We hypothesize that Ni-induced oxidative stress and disruption of iron hemostasis may have caused microcytic anemia in our study.

The fourth important finding of this study is that urine Cr was negatively associated with MCHC in the female participants. Cr is used in the tanning industry, paints, hip prosthetics and plating [31]. It causes pulmonary irritation, dermatological problems, renal damage and carcinogenicity [1]. Cr (VI) enters RBCs and is converted to Cr (III) by reducing glutathione, which is then excreted by the kidneys [32]. Cr (VI) is associated with high bioavailability and cellular toxicity [33]. Stana et al. conducted an animal study of 28 pregnant mice, in which the administration of Cr (VI) was found to be directly related to an increase in RBC membrane fragility [34]. Although Cr has been associated with RBC damage, few studies have investigated gender differences in Cr hemotoxicity. In the present study, we found that Cr was negatively associated with MCHC in the female participants. The mechanism behind the decreased MCHC in females needs further investigation. 

The fifth important finding of this study is that urine As was positively associated with MCV in the male participants. Arsenic poisoning by ingestion or inhalation manifests as bone marrow suppression, encephalopathy, neuropathy and carcinogenicity [35], and bone marrow suppression can lead to erythropoiesis dysfunction [36,37]. Previous studies have reported chronic As poisoning related to megaloblastic anemia [38]. In an animal study by Kannan et al., As exposure reduced the functional activities of RBC enzymes and decreased heme biosynthesis [39]. In terms of gender differences, Kile et al. conducted a study on 147 Bangladeshi women with As exposure and skin lesions and found a positive association between As exposure and anemia in women of reproductive age [40]. In the present study, we first found males’ MCV level was significantly associated with As. Further investigations are warranted to investigate if the gender dimorphism is affected by hormone or gender epigenetics.

Another important finding of this study is that urine Cd was positively associated with MCV in the male participants. Furthermore, the positive association between Cd and MCV was more pronounced in the male than in the female participants. Cd toxicity is well known in Asia due to events such as Cd poisoning of rice leading to itai-itai disease in Japan. Its toxicity causes anemia, osteoporosis, renal damage, pulmonary inflammation, carcinogenesis and impaired spermatogenesis [41]. Cd-induced anemia is caused by decreased erythropoietin production, iron deficiency and hemolysis due to RBC deformity [42]. In addition, Cd toxicity has been shown to decrease the glomerular filtration rate more significantly in females than in males [43], which causes the retention of Cd in the female body. The need to study gender differences in Cd poisoning was highlighted in a comparative study by Nishijo et al. in 2004, who found that gender differences in Cd toxicity were affected by distinct metabolism, kidney sensitivity and the efficiency of iron usage [44]. In the present study, we found a positive association between MCV and Cd exposure in the male participants. Since Cd affects both iron uptake and bone damage, macrocytic anemia could be explained by the carcinogenic properties of Cd outweighing its effects on iron uptake [45]. Hindrance of the effects of Cd by hypomethylating DNA on bone marrow differentiation can lead to macrocytic anemia. In addition, we found that the male participants were affected more significantly by Cd poisoning than the female participants. Although some studies have reported higher rates of Cd retention in females than in males [17,44], Fujiwara et al. found a decreased expression level of the iron transport gene in male mice [46]. We found a positive association between Cd and MCV in the male participants, and we hypothesize that impaired iron transport may affect erythropoiesis more than Cd retention.

The strength of this study is that we studied the impact of gender on heavy metal RBC hemogram parameters in a large population. However, there were also several limitations. First, only single measurements of metal concentrations were conducted. Second, as this was a cross-sectional study, causal relationships and long-term clinical outcomes could not be ascertained. Long-term prospective studies with serial heavy metal measurements in RBC hemogram changes are needed to verify our results. Third, as exposure was assessed using the total concentration of As in the urine. While such measurements can be performed quickly and are thus suitable when processing many samples, they do not reflect differences in metabolism and uptake between subjects. However, such measurements are considered to reasonably reflect exposure to inorganic As in clinical practice. Underestimation of As in terms of its effects on mortality was inevitable. Even though we measured blood and urinary concentrations of the heavy metals and obtained serum biochemical data, different exposure methods and routes may have different effects. Fourth, a lack of menopause data prevented us from discussing the effect of menopause in the female participants. Lastly, surveys were taken from the volunteer participants, mostly females, which could lead to selection bias and affect the interpretation of confidence intervals and standard errors. Though the majority of the participants were females, these participants were recruited by the advertisements posted in apartment complexes and sufficiently represented the middle class in the southern Taiwan population.

## 5. Conclusions

In conclusion, in this study, we analyzed a large cohort residing in southern Taiwan. The associations between heavy metals including Pb in the blood and Ni, Cr, Mn, As, Cu and Cd in the urine and RBC hemogram parameters were explored. The results show that in females, Pb and Ni were negatively associated with Hgb, MCV and MCHC, and Cr was also negatively associated with MCHC. In males, As and Cd were positively associated with MCV, and Pb was negatively associated with MCHC. In this study, heavy metal poisoning was found to disrupt hemogram parameters and was associated with gender differences.

## Figures and Tables

**Table 1:** Comparison of clinical characteristics among male and female participants.

Characteristics	All (*n* = 2447)	Male (*n* = 977)	Female (*n* = 1470)	*p*
Age (year)	55.1 ± 13.2	55.1 ± 13.6	55.1 ± 12.9	0.975
DM (%)	10.5	11.8	9.6	0.085
Hypertension (%)	25.3	28.2	23.4	0.007
BMI (kg/m^2^)	25.0 ± 3.9	25.5 ± 3.5	24.7 ± 4.1	<0.001
BMI group				<0.001
Underweight (<18.5)	2.7	1.4	3.3	
Normal weight (18.5–24.9)	50.3	43.6	50.8	
Overweight (25–29.9)	37.1	45.6	29.2	
Obese (≥30)	9.8	9.4	9.4	
SBP (mmHg)	132.1 ± 19.8	131.7 ± 17.5	132.3 ± 21.2	0.393
DBP (mmHg)	77.5 ± 11.7	79.0 ± 11.4	76.6 ± 11.8	<0.001
Laboratory parameters				
Fasting glucose (mg/dL)	99.9 ± 27.4	101.6 ± 29.8	98.7 ± 25.5	0.014
Triglyceride (mg/dL)	105 (73-150)	113 (80-174)	98 (68-140)	<0.001
Total cholesterol (mg/dL)	199.6 ± 37.4	193.8 ± 37.0	203.5 ± 37.2	<0.001
HDL cholesterol (mg/dL)	53.0 ± 13.6	46.8 ± 11.0	57.1 ± 13.7	<0.001
LDL cholesterol (mg/dL)	119.2 ± 34.0	118.8 ± 33.9	119.4 ± 34.1	0.648
Hgb (g/dL)	14.0 ± 1.6	15.1 ± 1.4	13.2 ± 1.3	<0.001
MCV (fl)	88.3 ± 7.3	88.9 ± 6.9	87.9 ± 7.6	0.001
MCHC (g/L)	33.0 ± 1.3	33.4 ± 1.2	32.7 ± 1.3	<0.001
eGFR (mL/min/1.73 m^2^)	89.1 ± 16.3	89.1 ± 12.9	89.1 ± 18.3	0.974
Uric acid (mg/dL)	5.7 ± 1.6	6.6 ± 1.5	5.1 ± 1.3	<0.001
Heavy metals				
Blood				
Pb (mg/L)	1.6 (1.0–2.2)	1.8 (1.2–2.6)	1.4 (0.9–2.0)	<0.001
Urine				
Ni (μg/L)	2.4 (1.5–3.7)	2.3 (1.5–3.4)	2.5 (1.6–3.9)	0.038
Cr (μg/L)	0.1 (0.1–0.1)	0.1 (0.1–0.1)	0.1 (0.1–0.1)	0.277
Mn (μg/L)	1.7 (0.9–3.0)	1.7 (0.9–2.9)	1.7 (0.9–3.0)	0.272
As (μg/L)	78.9 (45.6–142.0)	73.0 (45.3–125.3)	84.9 (46.2–151.5)	<0.001
Cu (μg/dL)	1.5 (1.0–2.0)	1.5 (1.1–2.0)	1.4 (1.0–1.9)	<0.001
Cd (μg/L)	0.8 (0.5–1.4)	0.7 (0.2–1.2)	0.9 (0.5–1.6)	<0.001

Abbreviations: As, arsenic; BMI, body mass index; Cd, cadmium; Cr, chromium; Cu, copper; DBP, diastolic blood pressure; DM, diabetes mellitus; eGFR, estimated glomerular filtration rate; HDL, high-density lipoprotein; Hgb, hemoglobin; LDL, low-density lipoprotein; MCHC, mean corpuscular hemoglobin concentration; MCV, mean corpuscular volume; Mn, manganese; Ni, nickel; Pb, lead; SBP, systolic blood pressure; WBC, white blood cell.

**Table 2:** Association of heavy metals and variables with Hgb using univariable linear regression analysis by gender.

Variables	Male		Female	
	Univariable		Univariable	
	Unstandardized Coefficient β (95% CI)	*p*	Unstandardized Coefficient β (95% CI)	*p*
Blood				
Pb (log per 1 mg/L)	−0.102 (−0.388, 0.185)	0.487	−0.136 (−0.402, 0.126)	0.306
Urine				
Ni (log per 1 μg/L)	−0.145 (−0.294, 0.004)	0.057	−0.286 (−0.399, −0.174)	<0.001
Cr (log per 1 μg/L)	−0.077 (−0.593, 0.439)	0.769	−0.109 (−0.553, 0.336)	0.631
Mn (log per 1 μg/L)	0.055 (−0.115, 0.224)	0.527	0.050 (−0.076, 0.176)	0.436
As (log per 1 μg/L)	−0.219 (−0.479, 0.041)	0.099	0.268 (0.088, 0.448)	0.003
Cu (log per 1 μg/dL)	−0.105 (−0.465, 0.256)	0.569	0.148 (−0.116, 0.412)	0.271
Cd (log per 1 μg/L)	−0.156 (−0.363, 0.050)	0.137	0.162 (0.005, 0.318)	0.043
Age (per 1 year)	−0.030 (−0.036, −0.024)	<0.001	0.009 (0.003, 0.014)	0.001
DM	−0.407 (−0.676, −0.139)	0.003	−0.321 (−0.554, −0.088)	0.007
Hypertension	−0.380 (−0.571, −0.188)	<0.001	0.038 (−0.124, 0.201)	0.642
BMI (per 1 kg/m^2^)	0.057 (0.032, 0.083)	<0.001	0.009 (−0.009, 0.026)	0.323
SBP (per 1 mmHg)	0.003 (−0.002, 0.008)	0.189	0.009 (0.005, 0.012)	<0.001
DBP (per 1 mmHg)	0.021 (0.013, 0.028)	<0.001	0.022 (0.016, 0.028)	<0.001
Triglyceride (log per 1 mg/dL)	1.245 (0.897, 1.593)	<0.001	0.966 (0.668, 1.264)	<0.001
Total cholesterol (per 1 mg/dL)	0.008 (0.005, 0.010)	<0.001	0.009 (0.007, 0.010)	<0.001
HDL cholesterol (per 1 mg/dL)	0.001 (−0.007, 0.009)	0.858	0.007 (0.002, 0.012)	0.007
LDL cholesterol (per 1 mg/dL)	0.007 (0.005, 0.010)	<0.001	0.008 (0.006, 0.010)	<0.001
eGFR (per 1 mL/min/1.73 m^2^)	0.033 (0.027, 0.040)	<0.001	0.001 (−0.002, 0.005)	0.471
Uric acid (per 1 mg/dL)	0.048 (−0.010, 0.106)	0.107	0.115 (0.063, 0.167)	< 0.001

Values expressed as unstandardized coefficient β and 95% confidence interval (CI). Abbreviations are the same as in Table 1.

**Table 3:** Association of heavy metals with Hgb using multivariable linear regression analysis by gender.

Heavy Metals	Male *		Female **		
	Multivariable		Multivariable		
	Unstandardized Coefficient β (95% CI)	*p*	Unstandardized Coefficient β (95% CI)	*p*	Interaction *p*
Blood					
Pb (log per 1 mg/L)	0.276 (−0.019, 0.572)	0.067	−0.429 (−0.698, −0.160)	0.002	0.826
Urine					
Ni (log per 1 μg/L)	−0.075 (−0.223, 0.072)	0.316	−0.271 (−0.378, −0.163)	<0.001	0.125
Cr (log per 1 μg/L)	0.106 (−0.385, 0.597)	0.672	−0.155 (−0.580, 0.271)	0.476	0.938
Mn (log per 1 μg/L)	0.082 (−0.084, 0.249)	0.331	0.060 (−0.061, 0.182)	0.330	0.946
As (log per 1 μg/L)	0.216 (−0.052, 0.484)	0.113	0.176 (−0.006, 0.359)	0.058	0.002
Cu (log per 1 μg/dL)	0.093 (−0.268, 0.454)	0.614	0.081 (−0.177, 0.338)	0.539	0.333
Cd (log per 1 μg/L)	0.092 (−0.117, 0.301)	0.389	0.102 (−0.051, 0.255)	0.190	0.016

Values expressed as unstandardized coefficient β and 95% confidence interval (CI). Abbreviations are the same as in Table 1. * Covariates in the multivariable model included age, diabetes, hypertension, body mass index, diastolic blood pressure, log triglyceride, total cholesterol and eGFR (significant variables in Table 2, but excluding LDL cholesterol due to multicollinearity). ** Covariates in the multivariable model included age, diabetes, systolic and diastolic blood pressures, log triglyceride, total cholesterol and uric acid (significant variables in Table 2, but excluding HDL cholesterol and LDL cholesterol due to multicollinearity).

**Table 4:** Association of heavy metals and variables with MCV using univariable linear regression analysis by gender.

Variables	Male		Female	
	Univariable		Univariable	
	Unstandardized Coefficient β (95% CI)	*p*	Unstandardized Coefficient β (95% CI)	*p*
Blood				
Pb (log per 1 mg/L)	−0.465 (−1.889, 0.959)	0.522	−1.643 (−3.126, −0.161)	0.030
Urine				
Ni (log per 1 μg/L)	−0.168 (−0.912, 0.576)	0.658	−1.288 (−1.922, −0.654)	<0.001
Cr (log per 1 μg/L)	−0.462 (−3.027, 2.104)	0.724	0.392 (−2.108, 2.892)	0.759
Mn (log per 1 μg/L)	0.034 (−0.809, 0.877)	0.937	0.182 (−0.528, 0.891)	0.616
As (log per 1 μg/L)	2.432 (1.147, 3.717)	<0.001	1.879 (0.871, 2.886)	<0.001
Cu (log per 1 μg/dL)	−0.288 (−2.061, 1.504)	0.752	0.158 (−1.327, 1.642)	0.835
Cd (log per 1 μg/L)	1.960 (0.940, 2.979)	< 0.001	0.529 (−0.353, 1.411)	0.240
Age (per 1 year)	0.075 (0.043, 0.106)	<0.001	0.111 (0.081, 0.140)	<0.001
DM	0.296 (−1.044, 1.635)	0.665	−0.293 (−1.606, 1.020)	0.662
Hypertension	0.323 (−0.635, 1.282)	0.508	0.701 (−0.212, 1.613)	0.132
BMI (per 1 kg/m^2^)	−0.056 (−0.184, 0.073)	0.396	−0.110 (−0.208, −0.012)	0.027
SBP (per 1 mmHg)	0.013 (−0.012, 0.037)	0.314	0.019 (0.001, 0.038)	0.038
DBP (per 1 mmHg)	−0.026 (−0.064, 0.012)	0.183	0.004 (−0.029, 0.037)	0.807
Triglyceride (log per 1 mg/dL)	−0.580 (−2.354, 1.193)	0.521	0.725 (−0.975, 2.425)	0.403
Total cholesterol (per 1 mg/dL)	0.002 (−0.010, 0.013)	0.765	0.025 (0.015, 0.036)	<0.001
HDL cholesterol (per 1 mg/dL)	0.038 (−0.002, 0.077)	0.060	0.061 (0.032, 0.089)	<0.001
LDL cholesterol (per 1 mg/dL)	0.002 (−0.011, 0.015)	0.745	0.016 (0.005, 0.027)	0.005
eGFR (per 1 mL/min/1.73 m^2^)	−0.055 (−0.089, −0.022)	0.001	−0.066 (−0.087, −0.046)	<0.001
Uric acid (per 1 mg/dL)	−0.133 (−0.422, 0.155)	0.364	0.224 (−0.071, 0.519)	0.137

Values expressed as unstandardized coefficient β and 95% confidence interval (CI). Abbreviations are the same as in Table 1.

**Table 5:** Association of heavy metals with MCV using multivariable linear regression analysis by gender.

Heavy Metals	Male *		Female **		
	Multivariable		Multivariable		
	Unstandardized Coefficient β (95% CI)	*p*	Unstandardized Coefficient β (95% CI)	*p*	Interaction *p*
Blood					
Pb (log per 1 mg/L)	−1.383 (−2.839, 0.073)	0.063	−4.492 (−6.091, −2.893)	<0.001	0.285
Urine					
Ni (log per 1 μg/L)	−0.406 (−1.148, 0.336)	0.283	−1.468 (−2.108, −0.828)	<0.001	0.013
Cr (log per 1 μg/L)	−0.515 (−3.053, 2.022)	0.690	0.065 (−2.364, 2.495)	0.958	0.976
Mn (log per 1 μg/L)	−0.095 (−0.931, 0.742)	0.825	−0.054 (−0.789, 0.680)	0.885	0.990
As (log per 1 μg/L)	1.462 (0.081, 2.842)	0.038	0.777 (−0.285, 1.840)	0.151	0.515
Cu (log per 1 μg/dL)	−0.662 (−2.460, 1.136)	0.470	−0.489 (−2.026, 1.048)	0.533	0.931
Cd (log per 1 μg/L)	1.297 (0.229, 2.364)	0.017	−0.206 (−1.115, 0.703)	0.657	0.030

Values expressed as unstandardized coefficient β and 95% confidence interval (CI). Abbreviations are the same as in Table 1. * Covariates in the multivariable model included age and eGFR (significant variables in Table 4). ** Covariates in the multivariable model included age, body mass index, systolic blood pressure, total cholesterol and eGFR (significant variables in Table 4, but excluding HDL cholesterol and LDL cholesterol due to multicollinearity).

**Table 6:** Association of heavy metals and variables with MCHC using univariable linear regression analysis by gender.

Variables	Male		Female	
	Univariable		Univariable	
	Unstandardized Coefficient β (95% CI)	*p*	Unstandardized Coefficient β (95% CI)	*p*
Blood				
Pb (log per 1 mg/L)	−0.948 (−1.195, −0.702)	<0.001	−1.177 (−1.425, −0.926)	<0.001
Urine				
Ni (log per 1 μg/L)	−0.077 (−0.210, 0.055)	0.252	−0.187 (−0.296, −0.077)	0.001
Cr (log per 1 μg/L)	−0.020 (−0.477, 0.438)	0.932	−0.490 (−0.920, −0.061)	0.025
Mn (log per 1 μg/L)	−0.057 (−0.207, −0.094)	0.460	0.066 (−0.056, 0.188)	0.289
As (log per 1 μg/L)	−0.086 (−0.316, 0.143)	0.461	0.072 (−0.101, 0.246)	0.413
Cu (log per 1 μg/dL)	−0.121 (−0.199, 0.440)	0.459	0.209 (−0.046, 0.464)	0.108
Cd (log per 1 μg/L)	−0.076 (−0.259, 0.107)	0.415	−0.064 (−0.215, 0.088)	0.411
Age (per 1 year)	−0.008 (−0.014, −0.003)	0.004	−0.001 (−0.006, 0.004)	0.771
DM	−0.035 (−0.274, 0.204)	0.772	0.064 (−0.162, 0.290)	0.577
Hypertension	−0.007 (−0.178, 0.164)	0.932	0.075 (−0.082, 0.232)	0.346
BMI (per 1 kg/m^2^)	0.021 (−0.001, 0.043)	0.067	0 (−0.016, 0.017)	0.960
SBP (per 1 mmHg)	0.010 (0.006, 0.015)	<0.001	0.002 (−0.001, 0.005)	0.187
DBP (per 1 mmHg)	0.010 (0.004, 0.017)	0.003	0.004 (−0.002, 0.010)	0.160
Triglyceride (log per 1 mg/dL)	0.724 (0.411, 1.037)	<0.001	0.474 (0.182, 0.765)	0.001
Total cholesterol (per 1 mg/dL)	0.001 (−0.001, 0.003)	0.317	0.003 (0.001, 0.005)	0.001
HDL cholesterol (per 1 mg/dL)	−0.008 (−0.015, −0.001)	0.019	0.002 (−0.003, 0.007)	0.380
LDL cholesterol (per 1 mg/dL)	0 (−0.002, 0.003)	0.685	0.002 (0, 0.004)	0.028
eGFR (per 1 mL/min/1.73 m^2^)	0.008 (0.002, 0.014)	0.011	0.001 (−0.003, 0.005)	0.612
Uric acid (per 1 mg/dL)	0.067 (0.016, 0.119)	0.010	0.094 (0.044, 0.145)	<0.001

Values expressed as unstandardized coefficient β and 95% confidence interval (CI). Abbreviations are the same as in Table 1.

**Table 7:** Association of heavy metals with MCHC using multivariable linear regression analysis by gender.

Heavy Metals	Male *		Female **		
	Multivariable		Multivariable		
	Unstandardized Coefficient β (95% CI)	*p*	Unstandardized Coefficient β (95% CI)	*p*	Interaction *p*
Blood					
Pb (log per 1 mg/L)	−0.902 (−1.154, −0.649)	<0.001	−1.311 (−1.568, −1.054)	<0.001	0.272
Urine					
Ni (log per 1 μg/L)	−0.042 (−0.173, 0.089)	0.530	−0.170 (−0.279, −0.061)	0.002	0.225
Cr (log per 1 μg/L)	−0.031 (−0.478, 0.416)	0.892	−0.483 (−0.909, −0.056)	0.027	0.245
Mn (log per 1 μg/L)	0.017 (−0.132, 0.166)	0.824	0.066 (−0.055, 0.187)	0.287	0.218
As (log per 1 μg/L)	0.011 (−0.234, 0.256)	0.930	0.046 (−0.131, 0.224)	0.607	0.285
Cu (log per 1 μg/dL)	0.117 (−0.202, 0.436)	0.470	0.211 (−0.047, 0.470)	0.109	0.779
Cd (log per 1 μg/L)	0.031 (−0.160, 0.221)	0.753	−0.059 (−0.211, 0.094)	0.451	0.901

Values expressed as unstandardized coefficient β and 95% confidence interval (CI). Abbreviations are the same as in Table 1. * Covariates in the multivariable model included age, systolic and diastolic blood pressures, log triglyceride, HDL cholesterol, eGFR and uric acid (significant variables in Table 6). ** Covariates in the multivariable model included log triglyceride, total cholesterol and uric acid (significant variables in Table 6, but excluding LDL cholesterol due to multicollinearity).

## Data Availability

Data may be available upon request to interested researchers. Please send data requests to: Szu-Chia Chen, Division of Nephrology, Department of Internal Medicine, Kaohsiung Medical University Hospital, Kaohsiung Medical University.
